# The Consumption of Beef Burgers Prepared with Wine Grape Pomace Flour Improves Fasting Glucose, Plasma Antioxidant Levels, and Oxidative Damage Markers in Humans: A Controlled Trial

**DOI:** 10.3390/nu10101388

**Published:** 2018-10-01

**Authors:** Inés Urquiaga, Danitza Troncoso, Maria José Mackenna, Catalina Urzúa, Druso Pérez, Sara Dicenta, Paula María de la Cerda, Ludwig Amigo, Juan Carlos Carreño, Guadalupe Echeverría, Attilio Rigotti

**Affiliations:** 1 Center for Molecular Nutrition and Chronic Diseases, School of Medicine, Pontificia Universidad Católica de Chile, Santiago 08330033, Chile; da.troncosos@gmail.com (D.T.); josemackenna@gmail.com (M.J.M.); catalina.urzua.u@gmail.com (C.U.); dperezp@uc.cl (D.P.); saradicenta@opalum.es (S.D.); pdelacerda@gmail.com (P.M.d.l.C.); gecheverria@bio.puc.cl (G.E.); arigotti@med.puc.cl (A.R.); 2 Department of Nutrition, Diabetes and Metabolism, School of Medicine, Pontificia Universidad Católica de Chile, Santiago 08330033, Chile; ludwig@med.puc.cl; 3 Agrosuper Comercializadora de Alimentos Ltda., Rancagua, Chile; jcarreno@agrosuper.com

**Keywords:** antioxidant, dietary intervention, fiber, oxidative damage, wine grape pomace

## Abstract

Wine grape pomace flour (WGPF) is a fruit byproduct that is high in fiber and antioxidants. We tested whether WGPF consumption could affect blood biochemical parameters, including oxidative stress biomarkers. In a three-month intervention study, 27 male volunteers, each with some components of metabolic syndrome, consumed a beef burger supplemented with 7% WGPF containing 3.5% of fiber and 1.2 mg gallic equivalents (GE)/g of polyphenols (WGPF-burger), daily, during the first month. The volunteers consumed no burgers in the second month, and one control-burger daily in the third month. At baseline and after these periods, we evaluated the metabolic syndrome components, plasma antioxidant status (i.e., 2,2-diphenyl-1-picrylhydrazyl radical scavenging capacity (DPPH), vitamin E, vitamin C), and oxidative damage markers (i.e., advanced oxidation protein products (AOPPs), oxidized low-density lipoproteins (oxLDLs), malondialdehyde (MDA)). The WGPF-burger intake significantly reduced glycemia and homeostatic model assessment-based measurement of insulin resistance. Vitamin C increased and decreased during the consumption of the WGPF-burger and control-burger, respectively. The WGPF-burger intake significantly decreased AOPP and oxLDL levels. Vitamin E and MDA levels showed no significant changes. In conclusion, the consumption of beef burgers prepared with WGPF improved fasting glucose and insulin resistance, plasma antioxidant levels, and oxidative damage markers. Therefore, this functional ingredient has potential as a dietary supplement to manage chronic disease risk in humans.

## 1. Introduction

Chronic non-communicable diseases are the main causes of disability and death in the developed and developing world. To prevent these diseases, a healthy lifestyle, including the intake of a salutogenic diet, is critical. The Mediterranean diet (MD) is considered to be a valuable preventive measure, since several studies have shown that its intake is associated with a lower incidence and prevalence of chronic diseases, and longer life expectancy in countries where an MD is routinely consumed or in subgroups exhibiting a higher score of MD adherence [1]. The MD is characterized by the primary consumption of olive oil, poultry, fish, fruits, vegetables, cereals, legumes, and nuts, moderate intake of fermented dairy products, such as yogurt and cheese, and low consumption of red meat [2]. Moderate intake of red wine with meals and cooking with herbs and spices also contribute to the healthy qualities of this diet [2]. 

In terms of nutrients, the MD is rich in monounsaturated fatty acids (MUFAs), complex carbohydrates, fiber, and antioxidants. It has a balanced ω-6/ω-3 polyunsaturated fatty acids (PUFAs) ratio and is low in saturated fat, refined carbohydrates, and terrestrial animal protein [3]. Conversely, the Western-type diet is defined by a high dietary intake of red meat, animal fat, dairy products, and sugar, and by a decreased consumption of legumes, vegetables, fruits, and seafood. Scientific evidence suggests that the high consumption of red and processed meats is associated with chronic disease conditions—especially cardiovascular and oncological diseases. Therefore, the current recommendation is the occasional consumption of red and processed meats [4]. Coincidentally, developed countries where populations consume an overall Western-type diet show a high risk of chronic diseases, so their diet has been catalogued as inconvenient and as a risk factor for these clinical conditions [5,6].

The negative effects of red and processed meat intake on chronic disease risk are attributed to their relatively high content of saturated fats, myoglobin, and nitrogenous preservatives [4]. With regard to myoglobin, this muscle protein contains iron, whose function is to store oxygen. Although it is essential for life, high consumption and excessive accumulation of iron are associated with oxidative damage and increased chronic disease risk [7,8,9]. Therefore, red meat consumption should be paired with the consumption of foods rich in antioxidants to counteract oxidative damage. From this evidence, it seems appropriate to develop reformulated meat products with a reduced sodium content, modified fatty acid profile, and added functional ingredients with beneficial health properties.

Despite growing evidence supporting an association between processed meat intake and chronic diseases, these foods continue to be consumed in high quantities, with an average intake of 13.7 kg/person in the United States [10] and 15.6 kg/person in Chile [11]. The magnitude of red meat consumption offers the opportunity to develop functional foods by adding fiber and antioxidants, which have positive effects on health, but are not consumed in sufficient quantities in most countries. Fiber and antioxidant intake is less than that recommended by the WHO [12], due to the low consumption of fruits, vegetables, and legumes. Indeed, these food groups, which are rich in antioxidants and fiber, are associated with a lower incidence of metabolic syndrome and chronic diseases [13,14]. Wine is also a source of polyphenol antioxidants and, when consumed in moderation, is associated with a reduced risk of cardiovascular diseases [15].

Traditionally, dietary fiber and antioxidants are addressed separately as unrelated dietary compounds. However, antioxidants (mainly polyphenols and carotenoids) are associated with dietary fiber, and are transported together along the small intestine [16]. These antioxidants are released from the fiber matrix within the colon by the action of bacterial microbiota, generating bioactive metabolites and an antioxidant environment [17]. Thus, it has been proposed that the transport of dietary antioxidants through the gastrointestinal tract may be an essential function of dietary fiber. Antioxidant dietary fiber is present in fruits, vegetables, and several derived products, such as grape and apple pomace [18]. In fact, some authors have prepared baked goods such as breads, muffins, and brownies with wine grape pomace flour (WGPF) as a source of antioxidant dietary fiber for promoting human health with good acceptance by consumers [19]. Thus, fruit byproducts represent rich sources of antioxidant dietary fiber and have beneficial bioactive properties. Furthermore, the use of these functional byproducts is eco-friendly and easily available in Chile.

We developed a foodstuff by enriching high-consumption red meat-based burgers with antioxidant dietary fiber obtained from WGPF [20]. We conducted an intervention study wherein volunteers with components of metabolic syndrome, a chronic condition with high cardiovascular and diabetic disease risk, consumed burgers with and without WGPF. The objective of this study was to test the effects of WGPF-burger consumption on components of metabolic syndrome, plasma antioxidants, and oxidative damage markers.

## 2. Materials and Methods

### 2.1. Test Burger Meal

WGPF was prepared from a cabernet sauvignon winemaking byproduct as described in Urquiaga et al. [20]. The WGFP contained 52% dietary fiber by dry weight, 41.11 ± 3.01 mg gallic equivalents (GE)/g of total polyphenols, 1.49 ± 0.18 mg/g cyanidin 3-glucoside equivalents of total anthocyanin, 362.9 μmol Trolox^®^ equivalents (TE)/g of oxygen radical absorbance capacity (ORAC), 53.51 ± 3.69 μg/g of α-tocopherol, 12.57 ± 0.71 μg/g γ-tocopherol, and 0.68 ± 0.07 μg/g of δ-tocopherol.

Beef burgers (100 g) were produced by Agrosuper Comercializadora de Alimentos Ltda. (Rancagua, Chile). Control-burgers without WGPF, and WGPF-burgers were made from the same raw beef and other components, and stored at −20 °C until use. The WGPF-burger was formulated with 7% of WGPF (Table 1).

### 2.2. Burger Antioxidant Composition Analysis

Representative samples (2 g) of burgers were homogenized in a 10 mL acetone/water/acetic acid (70:29.5:0.5) mixture using a tissue homogenizer for 30 s. The meat–liquid mixture was blended using a rotator at room temperature for 45 min, and centrifuged for 10 min at 3000 rpm. The supernatant fraction was kept, and the pellet was sequentially extracted two more times. All three supernatant fractions were combined and used to measure the total polyphenol and total anthocyanin content, and antioxidant capacity. Polyphenolic compounds were determined by the Folin–Ciocalteu procedure [21], total anthocyanin content was determined by the AOAC Official Method 2005.02, and antioxidant capacity was measured by the ORAC assay [22]. Tocopherol contents were determined using high-performance liquid chromatography (HPLC) with electrochemical detection according to Motchnik et al. [23]. Vitamin C (l-ascorbic acid) was analyzed using the high performance liquid chromatography (HPLC) method described by Kimoto et al. [24].

### 2.3. Subjects

A group of 34 male workers, ranging in age from 25 to 65 years, and who regularly consumed an omnivorous diet, participated in the study. All participants gave informed consent. The study was approved by the Ethics Committee of the School of Medicine at the Pontificia Universidad Católica de Chile (Code15-122) (ClinicalTrials.gov Identifier: NCT03592511).

Volunteers underwent a comprehensive medical history, physical examination, and clinical chemistry analysis before the study. The inclusion criteria were (1) having at least one of the five components of metabolic syndrome; and (2) a BMI (body mass index) between 25.0 and 39.9 kg/m^2^. The presence of metabolic syndrome components was defined using the criteria proposed by the Adult Treatment Panel III of the US National Cholesterol Education Program, which are (1) abdominal obesity as waist circumference >102 cm; (2) low levels (<40 mg/dL) of serum high-density lipoprotein (HDL) cholesterol; (3) hypertriglyceridemia (triglycerides ≥ 150 mg/dL); (4) elevated blood pressure (≥130/85 mmHg); and (5) fasting plasma glucose levels of 100 mg/dL or higher [25]. Exclusion criteria were the use of drug therapy for diabetes mellitus, hypertension or dyslipidemia, and the intake of pharmacological treatment with drugs that modify plasma antioxidant capacity or inflammation.

### 2.4. Study Design

The intervention was carried out at workplaces in Santiago, Chile. Workers were informed about the study and invited to participate. Initially, 34 male workers meeting all criteria agreed to partake, but only 27 workers completed the study. Seven volunteers quit the study because three disliked the blood sampling procedure, two were sent to work abroad, one left the workplace, and one presented gastrointestinal symptoms associated with WGPF-burger consumption.

The volunteers entered a longitudinal trial consisting of two treatment periods of four weeks, separated by a third four-week wash-out period. For the first four weeks, they consumed one WGPF-burger daily, then they were washed-out and, finally, they consumed one control-burger daily for four weeks. They were asked to maintain their regular eating habits and lifestyle during the study, except for the daily intake of burgers during the treatment periods. The burgers were eaten as a replacement for red or processed meat consumption, or in addition to their regular meal when it did not contain meat products. During the washout period, all subjects consumed their usual diet. Burger intake was supervised every day at lunch at the canteens of the workplaces. On weekends, participants were asked to consume burgers at home with their regular meals. In addition, compliance with burger consumption was carefully monitored by frequent calls from the dietitian.

Blood samples were obtained at weeks 0, 4, 8, and 12 for analysis. Participants had clinical, nutritional, and anthropometric evaluations at the beginning and end of the study.

### 2.5. Anthropometric and Blood Pressure Measurements

Height and weight were recorded at the start and end of the intervention period. Systolic and diastolic blood pressures were measured on the left arm at heart level after at least 5 min of resting in a sitting position, using a mercury sphygmomanometer. Two readings, separated by at least 1 min, were taken, and the mean value was calculated and recorded. If there was more than a 5 mmHg difference between the first and second readings, an additional reading was obtained and used to calculate the recorded mean value [26].

### 2.6. Mediterranean Diet Score

Overall food intake was evaluated using a self-reported questionnaire with fourteen items that measured adherence to the Mediterranean diet in Chile, the Chilean-MDI [27]. This scale was designed based on traditional food consumption habits in Mediterranean countries, with selective modifications to incorporate Chilean dietary habits. Scores ranged from 0 (minimal adherence) to 14 (maximal adherence).

### 2.7. Biochemical Procedures

Venous blood samples were taken after a 12 h fasting period and collected in heparin, citrate, and anticoagulant-free BD Vacutainer^®^ tubes. Glucose, albumin, uric acid, bilirubin, creatinine, calcium, total cholesterol, HDL-cholesterol, LDL-cholesterol, triglycerides, glutamic-oxaloacetic transaminase, gammaglutamyl transpeptidase, and alkaline phosphatase were measured in serum using a spectrophotometer autoanalyzer (Hitachi 917; Roche Diagnostics^®^, Branchburg, NJ, USA) with reagent kits purchased from the manufacturer. Insulin was measured by electrochemiluminescence immunoassay (ECLIA) (Roche Diagnostics^®^).

To determine vitamin C levels, blood samples kept on ice were analyzed the same day that blood was drawn. l-Ascorbic acid was determined by spectrophotometry using a multi-detection microplate reader, Synergy HT (BIO-TEK, Montpelier, VT, USA) [23].

Tocopherols (i.e., α-tocopherol, γ-tocopherol, and δ-tocopherol) were determined in plasma using HPLC with electrochemical detection according to Motchnik et al. [23].

Total plasma antioxidant capacity was evaluated by total radical trapping potential (TRAP) [28] and 2,2-diphenyl-1-picrylhydrazyl (DPPH) [29] assays. Trolox^®^ (6-hydroxy-2,5,7,8-tetramethylchroman-2-carboxylic acid) was chosen as the standard antioxidant.

Advanced oxidation protein products (AOPPs) were determined in the plasma using the method described by Witko-Sarsat et al. [30]. Malondialdehyde (MDA) levels were quantified according to Templar et al. [31]. Oxidized low-density lipoprotein (oxLDL) was determined by the oxLDL/MDA Adduct Enzyme-Linked ImmunoSorbent Assay (ELISA) Kit (Immundiagnostik, AG, Bensheim, Germany).

### 2.8. Statistical Analysis

Variables were classified as parametric (normal distribution) or non-parametric for all statistical analyses. Parametric continuous variables were presented with means and standard deviations, while non-parametric variables were presented with medians and ranges. Categorical variables were presented as the number of cases and percentages.

To evaluate changes between the 4-week intervention periods over time, parametric biochemical measurements (MDA, AOPP, TRAP, DPPH, vitamin C, and vitamin E) were assessed by analysis of variance (ANOVA) of four repeated measures with the repeated contrast method. For non-parametric variables (oxLDL), the Friedman test was used.

Data processing and statistical analyses were performed using the statistical software package SPSS, version 17.0 (SPSS Inc., Chicago, IL, USA). A *p*-value ≤ 0.05 was considered statistically significant.

## 3. Results

### 3.1. Burger Composition

Table 1 shows the composition of burgers used in the study. The raw WGPF-burger contained 3.5% fiber, 1.2 mg GE/g of polyphenols, and 17.2 µmol TE/g of ORAC. The raw control-burger contained no fiber, 0.396 mg GE/g of polyphenols, and 1.82 µmol TE/g of ORAC.

### 3.2. Subject Baseline Characteristics

Twenty-seven participants completed the protocol, which was developed between May and November 2016. Table 2 shows the anthropometric, clinical, and biochemical baseline parameters. Only 7.4% (*n* = 2) of participants had a normal weight, and 48.1% (*n* = 13) and 44.4% (*n* = 12) were overweight and obese, respectively. Initially, 25.9% (*n* = 7) of volunteers had metabolic syndrome. Analyzing its components, 51.9% (*n* = 14) of volunteers had abdominal obesity; 37.0% (*n* = 10) had high triglycerides; 29.6% (*n* = 8) had low HDL cholesterol; 40.7% (*n* = 11) were hypertensive; and 7.4% (*n* = 2) exhibited hyperglycemia.

With regard to overall diet quality intake, the Chilean-MDI of the volunteers had a mean score of 4.99 ± 1.91 points. More than half (55.6%) of the group had a modestly Mediterranean diet adherence (5 to 8.5 points), while 44.4% had an unhealthy score (0 to 4.5 points). None of the participants had a diet score that qualified as healthy or high Mediterranean diet adherence (9 to 14 points).

### 3.3. Anthropometric Measurements and Metabolic Syndrome Component Prevalence over Time

The anthropometric features of the group did not change significantly over the course of the intervention period including no variations in BMI or number of subjects with normal weight, overweight, and obesity. During the study, no significant changes were observed in percentages of subjects presenting abdominal obesity, high triglycerides, low HDL cholesterol, high blood pressure, high glucose, and metabolic syndrome. Additionally, total cholesterol and LDL cholesterol did not change significantly during the study. The Chilean-MDI score did not vary significantly from the beginning and the end of the study (4.94 ± 1.69 points at study completion). At the end of the study, 3.8%, 46.2%, and 50.0% of participants had diet quality scores categorized as healthy, moderately healthy, and unhealthy, respectively.

### 3.4. Effect of Consuming Burgers on Plasma Glucose and Insulin Levels

As high plasma glucose levels are a component of metabolic syndrome, we evaluated the effect of consuming the WGPF-burger and control-burger on glycemia and plasma insulin, and calculated the HOMA (homeostatic model assessment) index—a reliable and clinically useful indicator of insulin resistance.

**Figure 1:**
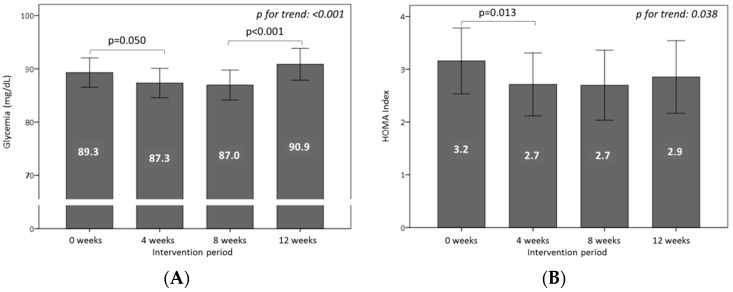
Effects of the consumption of burgers on (**A**) glycemia and (**B**) HOMA (homeostatic model assessment) throughout the study. Volunteers consumed one WGPF-burger daily between weeks 0 and 4 (intervention period), followed by a washout period (weeks 4 to 8), and then consumed one control-burger daily between weeks 8 and 12 (control period). Bars represent the mean value for each time. Lines represent the 95% confidence interval. Analysis of variance (ANOVA) of four repeated measures with the repeated contrast method was used to evaluate changes between the 4-week intervention periods over time.

Figure 1A shows the plasma glucose levels during the study. Glycemia decreased significantly (*p* = 0.050) during the WGPF-burger period, and remained low throughout the washout period. By contrast, the intake of the control-burger increased plasma glucose significantly (*p* < 0.001). On the other hand, plasma insulin levels did change, but differences were not statistically significant (*p* = 0.055) (not shown). Plasma insulin displayed a decrease trend during the WGPF-burger period (−13%) and remained lower during the washout period and after the control-burger period. Figure 1B shows the HOMA index values throughout the study. HOMA decreased significantly (*p* = 0.013) during WGPF-burger consumption, and remained low during the washout and control-burger periods.

### 3.5. Effect of Consuming Burgers on Antioxidants and the Total Antioxidant Capacity in Plasma

**Figure 2:**
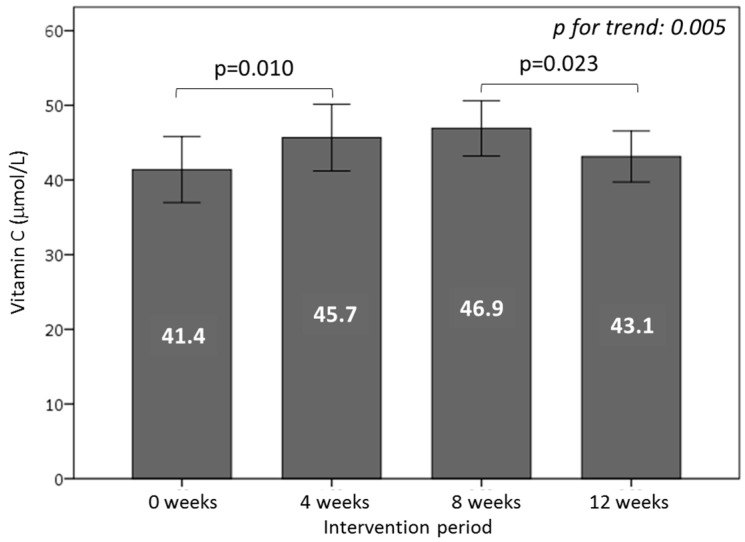
Effect of the consumption of burgers on plasma vitamin C throughout the study. Volunteers consumed one WGPF-burger daily between weeks 0 and 4 (intervention period), followed by a washout period (weeks 4 to 8), and then consumed one control-burger daily between weeks 8 and 12 (control period). Bars represent the mean value for each time; lines represent the 95% confidence interval. Analysis of variance (ANOVA) of four repeated measures with the repeated contrast method was used to evaluate changes between the 4-week intervention periods over time.

To evaluate the differential effect of both types of burgers on plasma antioxidants, we measured blood levels of vitamin C, total vitamin E, α- and γ-tocopherols, and uric acid. The consumption of the WGPF-burger increased plasma vitamin C (*p* = 0.010), an essential contributor to plasma antioxidant defense, whereas the control-burger intake decreased its concentration (*p* = 0.023) to baseline levels (Figure 2). During the washout period, no changes in antioxidant levels were observed. By contrast, the total plasma concentrations of vitamin E, α-tocopherol, and γ-tocopherol, key fat-soluble antioxidants, did not change significantly during the study.

**Figure 3:**
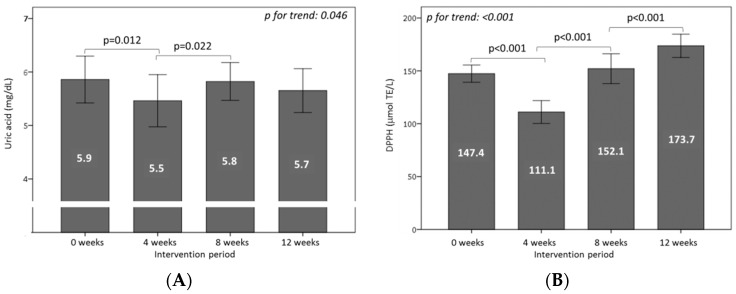
Effect of the consumption of burgers on (**A**) uric acid and (**B**) antioxidant capacity in plasma throughout the study. Volunteers consumed one WGPF-burger daily between weeks 0 and 4 (intervention period), followed by a washout period (weeks 4 to 8), and then consumed one control-burger daily between weeks 8 and 12 (control period). Bars represent the mean value for each time; lines represent the 95% confidence interval. Analysis of variance (ANOVA) of four repeated measures with the repeated contrast method was used to evaluate changes between the 4-week intervention periods over time. DPPH: 2,2-diphenyl-1-picrylhydrazyl; TE: Trolox^®^ equivalent.

Plasma uric acid is an important contributor to total plasma antioxidant capacity and may have a physiological function in counteracting oxidative damage. Plasma uric acid levels decreased significantly with WGPF-burger consumption (*p* = 0.012). During the washout period, plasma uric acid recovered to the baseline concentrations (*p* = 0.022), and remained steady during the control-burger consumption period (Figure 3A).

To determine the effect of burger consumption on plasma antioxidant activity, TRAP and DPPH radical scavenging capacity were evaluated. Of these two measurements, only the DPPH antioxidant assay changed significantly over the course of the study, diminishing during WGPF-burger consumption (*p* < 0.001). After the washout (*p* < 0.001) and control-burger intake (*p* < 0.001) periods, DPPH radical activity rose significantly (Figure 3B).

### 3.6. Effect of Consuming Burgers on Oxidative Damage Markers in Plasma

**Figure 4:**
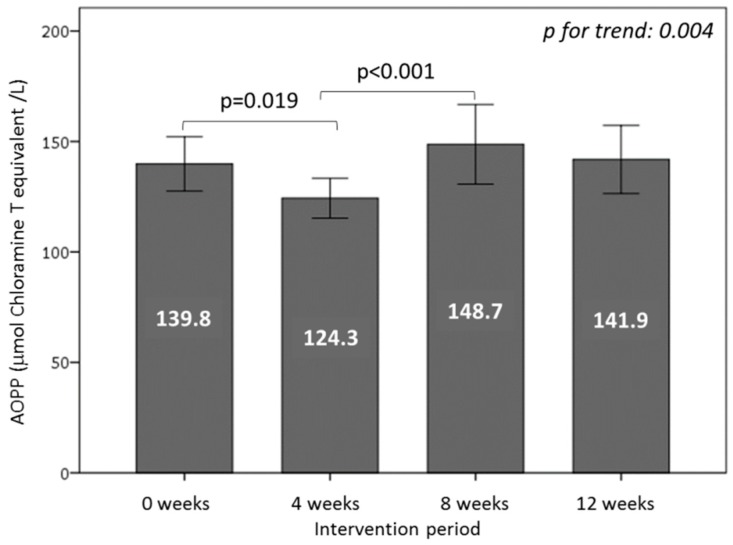
Protein oxidative damage measured as plasma advanced oxidation protein product (AOPP) concentrations of volunteers during the study. Volunteers consumed one WGPF-burger daily between weeks 0 and 4 (intervention period), followed by a washout period (weeks 4 to 8), and then consumed one control-burger daily between weeks 8 and 12 (control period). Bars represent the mean value for each time; lines represent the 95% confidence interval. Analysis of variance (ANOVA) of the four repeated measures with the repeated contrast method was used to evaluate changes between the 4-week intervention periods over time.

**Figure 5:**
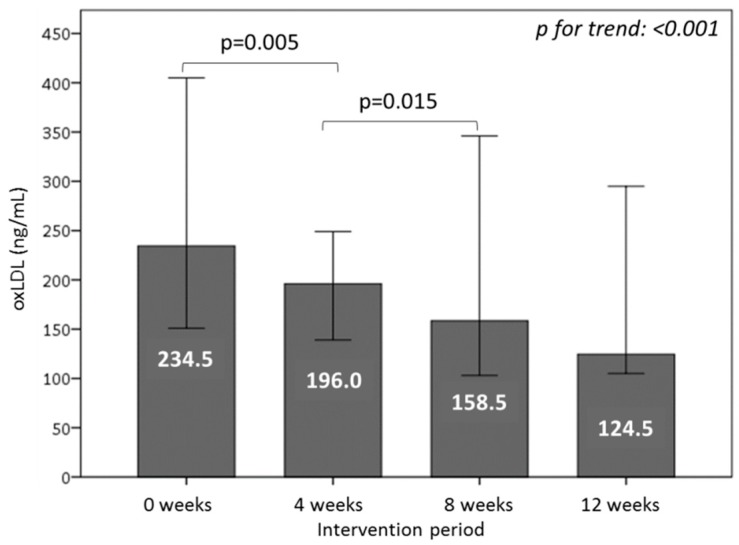
Oxidative damage to LDL, measured as plasma oxLDL concentrations of volunteers during the study. Volunteers consumed one WGPF-burger daily between weeks 0 and 4 (intervention period), followed by a washout period (weeks 4 to 8), and then consumed one control-burger daily between weeks 8 and 12 (control period). Bars represent the mean value for each time; lines represent the 95% confidence interval. The Friedman test was used to evaluate changes between the 4-week intervention periods over time. LDL: low-density lipoprotein; oxLDL: oxidized low-density lipoprotein.

To determine the effect of WGPF-burger and control-burger consumption on oxidative damage markers, we measured the MDA, AOPP, and oxLDL. MDA showed a decrease trend during the WGPF-burger period (−6%) and slightly increased in the washout period (5%), but the changes were not statistically significant. After the control-burger period, no further modifications were observed. In addition, AOPP levels decreased significantly after the consumption of WGPF burgers (*p* = 0.019). In the washout period, AOPP returned to baseline levels (*p* < 0.001), and remained high during the control-burger intake period (Figure 4). Finally, the oxLDL levels diminished significantly after WGPF-burger consumption (*p* = 0.005) and washout periods (*p* = 0.015), and remained low after the control-burger period (Figure 5).

## 4. Discussion

In this study, we developed a foodstuff by enriching red meat-based burgers with antioxidant dietary fiber obtained from wine grape pomace flour (WGPF) [20]. The WGPF was made from seed and skin residues obtained from grapes during winemaking, and contained high levels of phenolic compounds and dietary fiber.

Throughout this short-term study, there were no significant changes in the prevalence of subjects presenting with abdominal obesity, high triglycerides, low HDL cholesterol, high blood pressure, high glucose, high total and LDL cholesterol, or metabolic syndrome. In addition, BMI did not change significantly. These findings agreed with the fact that the group did not modify its overall food consumption habits as measured with the Chilean-MDI score.

However, a significant reduction in fasting glycemia was observed after four weeks of WGPF-burger consumption. The formulation used in this study contained 7 g of WGPF, suggesting that this amount per day was sufficient to reduce fasting glucose levels. In a previous study, where volunteers consumed 20 g of WGPF per day over 16 weeks, a statistically significant decrease in fasting glycemia was also found when compared with the control group [20]. There is evidence that dietary fiber consumption is associated with reduced food glycemic index, improved glucose metabolism, prevention or delayed progression of impaired glucose tolerance and insulin resistance to diabetes, and lower fasting glucose levels in diabetic individuals [32]. Nevertheless, the mechanisms associated with these beneficial effects are not yet known [33]. Still, it is not clear whether fiber—with its associated antioxidants—is the main component responsible for lowering glucose, or if there is another constituent in WGPF that contributes to this effect [34]. However, we cannot discard that this finding may have been caused by the interaction of meat and WGPF. This formulation may have altered the release and/or absorption of a biologically active component from WGPF that improved glucose homeostasis [35].

In addition, there was a statistically significant decrease in the HOMA index after the WGPF-burger consumption period, but the observed reduction in fasting insulin concentrations was not statistically significant (*p* = 0.055). This effect, concomitant with the reduction in glycemia, suggests an increase in insulin sensitivity. During the washout period, glycemia, insulin levels, and the HOMA index remained constant. This finding may be explained by a residual WGPF-burger effect or by a slow mechanism of adaptation. The consumption of control-burgers correlated with return of glycemia to its initial values, but did not affect the HOMA index and plasma insulin. Thus, the HOMA index seems to require more time to return to basal levels, as the insulin levels were persistently lower. Another intervention trial showed a statistically significant difference in postprandial insulin concentration variations, measured by an oral glucose test, after 16 weeks of the consumption of 20 g WGPF per day [20]. In addition, a whole grain cereal-based diet significantly lowered postprandial plasma insulin concentrations in individuals with metabolic syndrome [36]. It is likely that bioactive compounds in this fiber-enriched ingredient, such as the fiber itself, trace minerals, phenolic antioxidants, vitamin E, or phytoestrogens, may contribute to improving insulin sensitivity through underlying mechanisms that remain to be established [37].

During gastric digestion, reactive oxygen species are generated from lipid rich-foods, leading to enhanced lipid peroxidation, oxidation of the amino acid side chains present in dietary proteins, the formation of protein–protein cross-linkages, protein backbone damage resulting in protein fragmentation [38], and vitamin co-oxidation [39]. These oxidized species are absorbed from the gastrointestinal tract into the blood, and some of them consume plasma antioxidants [40]. In particular, red meat consumption is associated with post-prandial oxidative stress, which is characterized by an increase in oxidative stress markers, such as blood hydroperoxides [40]. Many of these products, which are generated from lipid peroxidation in the stomach, are absorbed by the intestine and interact with proteins and lipids to form advanced lipid oxidation end-products (ALEs) and AOPPs [41,42]. Together with the findings using the control-burger in this study, these observations indicate that the oxidative stress caused by the intake of red meat without concomitant antioxidant supplementation reduces the overall antioxidant defenses in the body, and may partly explain the association between red meat consumption and chronic diseases [43].

Vitamin C is an essential nutrient with antioxidant properties, and is an important contributor to plasma antioxidant capacity. Moreover, vitamin C is involved in the regeneration of circulatory antioxidant molecules including glutathione, α-tocopherol, urate, and β-carotene, based on its ability to reduce oxidized species. Many epidemiological studies have found an association between chronic vitamin C deficiency and increased risk of developing a range of age-associated conditions, such as cardiovascular disease, type 2 diabetes, and cancer [44]. Within this context, the intake of WGPF rich in antioxidant capacity may reduce the generation of oxidant species during the gastric phase of digestion [45]. Consequently, the absorption of oxidized species, especially lipoperoxides, from the gastrointestinal tract into the blood, would also likely decrease, and the vitamin C spent would be lower. Indeed, a significant increase in plasma vitamin C concentrations was observed after four weeks of WGPF-burger consumption. In contrast, after control-burger consumption, the vitamin C levels decreased as higher oxidized species are generated during gastric digestion and absorbed, leading to plasma vitamin C consumption as a consequence of reducing oxidized species.

Plasma uric acid, a contributor of plasma antioxidant capacity, has been proposed as another key physiological responder against oxidative injury, and increases in its plasma concentration occur during oxidative stress in humans [46,47]. A significant decrease in uric acid levels and DPPH radical scavenging capacity was observed after four weeks of WGPF-burger consumption. We hypothesized that the plasma uric acid levels and antioxidant capacity diminished during WGPF-burger consumption because WGPF reduced oxidative injury, as shown by the AOPP and oxLDL analyses, decreasing uric acid demand and utilization. Additionally, uric acid and AOPP showed similar changes during the study, however, vitamin C decreased later, suggesting a different mechanism of regulation for both antioxidants and the participation of other compounds or enzymes.

Advanced oxidation protein products (AOPPs) promote oxidative stress and inflammation, thus, they may participate in many pathophysiological processes involved in chronic diseases [48]. The formation of AOPPs is irreversible, and they cannot be easily hydrolyzed by proteolytic enzymes or reduced by antioxidants such as vitamin C and glutathione [49]. AOPPs are mostly eliminated by the liver and spleen [50,51,52]. In addition to chronic uremia, AOPP accumulation has been found in patients with atherosclerosis, coronary artery disease, diabetes mellitus, systemic sclerosis, chronic rheumatic valve disease, and colorectal cancer, among other chronic conditions [53,54]. In our study, we observed a reduction in plasma AOPP concentrations after WGPF-burger consumption, which returned to baseline levels following the washout period. AOPP concentrations remained steady during the control-burger period. The WGPF-dependent changes observed in the AOPP plasma levels may be explained by lower production and enhanced elimination of these oxidation products.

In this study, we also observed a decrease in oxLDL during the WGPF-burger period, together with changes in AOPP plasma concentrations. There is some evidence that AOPP and oxidized lipoproteins act in concert in the development of atherosclerosis [55]. AOPP-LDL can be found within the atherosclerotic vessel walls and plaques in chronic renal failure patients [56,57]. Moreover, circulating oxLDL concentrations have been recognized as a risk factor for cardiovascular disease, and seem to play a critical role in atherogenesis [58]. WGPF is rich in macromolecular antioxidants that could counteract the production of oxidant molecules that react with LDL and generate oxLDL. Based on the available evidence, a healthy diet such as the MD, that includes a combination of antioxidant compounds and flavonoid-rich foods, appears to effectively decrease LDL particle oxidizability, which may give some insight into its cardiovascular benefits.

Consistent with other studies, regarding the deleterious effects of hyperglycemia and oxidative stress in subjects with impaired fasting glucose when compared with those with normal fasting glucose [59], the present study showed consistency between the glycemic changes, plasma antioxidants, and oxidative damage caused by the change in diet. To determine if these variations have long-term clinical significance, more studies would be required in patients with some chronic disease (e.g., diabetics).

The major strengths of the current study were the application of a standardized and homogenous formulation of this functional food, as well as the daily supervision of WGPF-burger and control-burger intake during lunch, as they were offered and consumed at workplace cafeterias. The limitations of the study were the reduced number of participants who completed the intervention protocol and the duration of the study. In addition, this intervention was an open-labelled trial that may have led to potential biases in the subjects as well as in the researchers. Thus, it would be interesting to perform a longer double-blinded study in a greater number of subjects with high chronic disease risk to corroborate our findings, including additional relevant clinical outcomes.

## 5. Conclusions

The consumption of a beef burger with WGPF—a functional ingredient rich in macromolecular antioxidants and fiber—improved glycemia, enhanced insulin sensibility, and decreased oxidative stress, as indicated by increased antioxidant defenses and reduced oxidative damage markers. Given that these biomarkers are associated with chronic diseases, WGPF is an ingredient with nutritional potential for the prevention and/or treatment of chronic conditions, such as diabetes mellitus and cardiovascular disease.

## Figures and Tables

**Table 1:** Composition of burgers.

	WGPF-Burger (100 g)	Control-Burger (100 g)
**Energy (kcal)**	168	168
**Proteins (g)**	13.3 ± 0.16	16.0 ± 0.24
**Total fats (g)**	10.9 ± 0.02	11.6 ± 0.06
**Saturated fatty acids (g)**	5.7 ± 0.04	5.9 ± 0.07
**Trans fatty acids (g)**	0.0	0.0
**Monounsaturated fatty acids (g)**	5.0 ± 0.04	5.3 ± 0.05
**Polyunsaturated fatty acids (g)**	0.2 ± 0.05	0.2 ± 0.05
**Cholesterol (mg)**	81 ± 4	81 ± 4
Available carbohydrates * (g)	4.1 ± 0.67	0.0
**Total sugars (g)**	0.1	0.0
**Fiber (g)**	3.5 ± 0.67	0.0
**Polyphenols (mg GE/g)**	1.21 ± 0.02	0.396 ± 0.019
**ORAC (μmol TE/g)**	17.2 ± 1.29	1.82 ± 0.23

Data were expressed as the mean ± standard deviation (SD). GE: gallic equivalent; ORAC: oxygen radical absorbance capacity; TE: Trolox^®^ equivalent; WGPF: wine grape pomace flour. * Nitrogen-free extract minus dietary fiber.

**Table 2:** Baseline anthropometric, clinical, and biochemical characteristics of volunteers (*n* = 27).

Parameter	Mean ± SD
Age (years)	43.6 ± 11.2
***Anthropometric parameters***	
Weight (kg)	87.1 ± 13.5
Body mass index (kg/m^2^)	29.5 ± 3.7
Waist circumference (cm)	101.0 ± 9.6
***Blood pressure***	
Systolic blood pressure (mmHg)	122.1 ± 15.1
Diastolic blood pressure (mmHg)	80.2 ± 10.6
***Glucose metabolism***	
Glucose (mg/dL)	89.3 ± 7.0
Insulin (mg/dL)	14.2 ± 6.8
***Lipid profile***	
Total cholesterol (mg/dL)	189.8 ± 30.5
LDL cholesterol (mg/dL)	109.7 ± 31.1
HDL cholesterol (mg/dL)	47.3 ± 14.7
Triglycerides (mg/dL)	163.3 ± 133.9

HDL: high-density lipoprotein; LDL: low-density lipoprotein.
